# Different patterns of spreading direction and motor neurons involvement in a cohort of limb‐onset amyotrophic lateral sclerosis patients from Southern Italy: Potential implication on disease course or progression?

**DOI:** 10.1002/brb3.2899

**Published:** 2023-05-19

**Authors:** Giammarco Milella, Stefano Zoccolella, Daniele Urso, Salvatore Nigro, Ludovica Tamburrino, Valentina Gnoni, Marco Filardi, Giancarlo Logroscino

**Affiliations:** ^1^ Department of Basic Medicine, Neuroscience and Sense Organs University of Bari Aldo Moro Bari Italy; ^2^ ASL Bari San Paolo Hospital, Neurology Unit Milan Italy; ^3^ Center for Neurodegenerative Diseases and the Aging Brain University of Bari Aldo Moro at Pia Fondazione “Card. G. Panico,” Tricase Italy

**Keywords:** amyotrophic lateral sclerosis, prognostic, spreading directions

## Abstract

**Background:**

Currently, there is a lack of knowledge concerning where the pathological process starts and how the neurodegeneration spreads during the course of amyotrophic lateral sclerosis (ALS).

**Aims:**

This study aims to evaluate the spreading direction of the disease and the corresponding clinical characteristics in a cohort of patients with limb‐onset ALS.

**Patients and methods:**

Consecutive incident ALS patients referring to an ALS tertiary center from Southern Italy, between 2015 and 2021, were recruited in the study. According to the initial directions of spread, patients were dichotomized into horizontal spreading pattern (HSP) or vertical spreading pattern (VSP) groups.

**Results:**

Among 137 newly diagnosed ALS, 87 presented a spinal onset. Ten patients with pure LMN were not included in the study. All cases reported a clear spread direction. The frequency of HSP and VSP spreading was similar overall (47 vs. 30). The prevalence of HSP was higher (74% vs. 50%) in patients with upper limb‐onset (UL‐ALS), compared to patients with lower limb‐onset (LL‐ALS; *p* < .05). Conversely, the occurrence of VSP spread was threefold higher in patients with LL‐ALS, compared to UL‐ALS (*p* < .05). Patients with VSP showed a wider upper motor neuron impairment, whereas the involvement of LMN resulted greater in patients with HSP. Patients with HSP exhibited a greater drop of ALSFRS‐r sub‐score in the region of onset, while VSP showed a slighter but more diffuse reduction of ASLFRS‐r subscore in more body districts beyond the site of onset. Patients with VSP were also characterized by a higher median progression rate and an earlier median bulbar involvement, compared to HSP.

**Conclusions:**

Our findings suggested investigating the spreading direction of ALS among patients with spinal onset, to better delineate the clinical profiles of patients with ALS, and predict an earlier impairment of bulbar muscle and a more rapid progression of the disease

## Graphical abstract

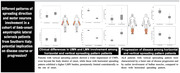

## 1 BACKGROUND

Amyotrophic lateral sclerosis (ALS) is a fatal neurodegenerative disorder characterized by progressive degeneration of motor neurons in the cortex, the brainstem, and the spinal cord (Kiernan et al., 2011). Heterogeneous motor manifestations are caused by simultaneous and progressive degeneration of upper motor neurons (UMNs) and lower motor neurons (LMNs) (Kiernan et al., 2011). Currently, through the available clinical and instrumental exams, it is still impossible to reveal if the pathological process starts in the motor neurons of the cortex, brainstem, or spinal cord. This latter limit is due to the lack of studies performed during the pre‐symptomatic or very early phases of the disease. Indeed, patients with ALS commonly refer to a tertiary center after a median interval from symptom onset of 12 months (Zoccolella et al., 2006), during which simultaneous and progressive degeneration of UMNs and LMNs occur. Therefore, all the information regarding the primary pathologic focus and spreading pattern is based on the anamnestic history reported by patients during their first visit.

According to available population‐based studies, the first symptoms occur in only one body region in 90%−98% of cases (Ravits et al., 2007; Turner et al., 2010). The subsequent progression is either confined largely to the same region (e.g., cervical or lumbosacral districts) before the involvement of other body districts or has a more diffuse spreading, with early and simultaneous involvement of several regions. Spread seems to be preferentially contiguous in either a horizontal, rostral‐caudal, or caudal‐rostral direction (Gargiulo‐Monachelli et al., 2012; Körner et al., 2011; Ravits & La Spada, 2009).

In 2007, Ravits et al. (2007) reviewed the clinical history of 100 patients with ALS and found that both UMN and LMN signs spread outward to contiguous regions following the underlying, somatotopically arranged, neuronal anatomy. This is best seen when the onset of symptoms is spinal. For example, LMN clinical deficits may progress from one arm to the other, consistently with the neuronal anatomy of LMNs in the spinal cord. Instead, UMN clinical deficits progress from one arm to the ipsilateral leg, consistent with the somatotopic anatomy of the cerebral cortex (Ravits et al., 2007).

Based on this postulation, we studied a cohort of consecutive patients with limb‐onset ALS to evaluate the spreading direction of motor symptoms of the disease and the impact of the spreading direction on clinical characteristics.

## 2 METHODS

### 2.1 Subjects and inclusion/exclusion criteria

**FIGURE 1:**
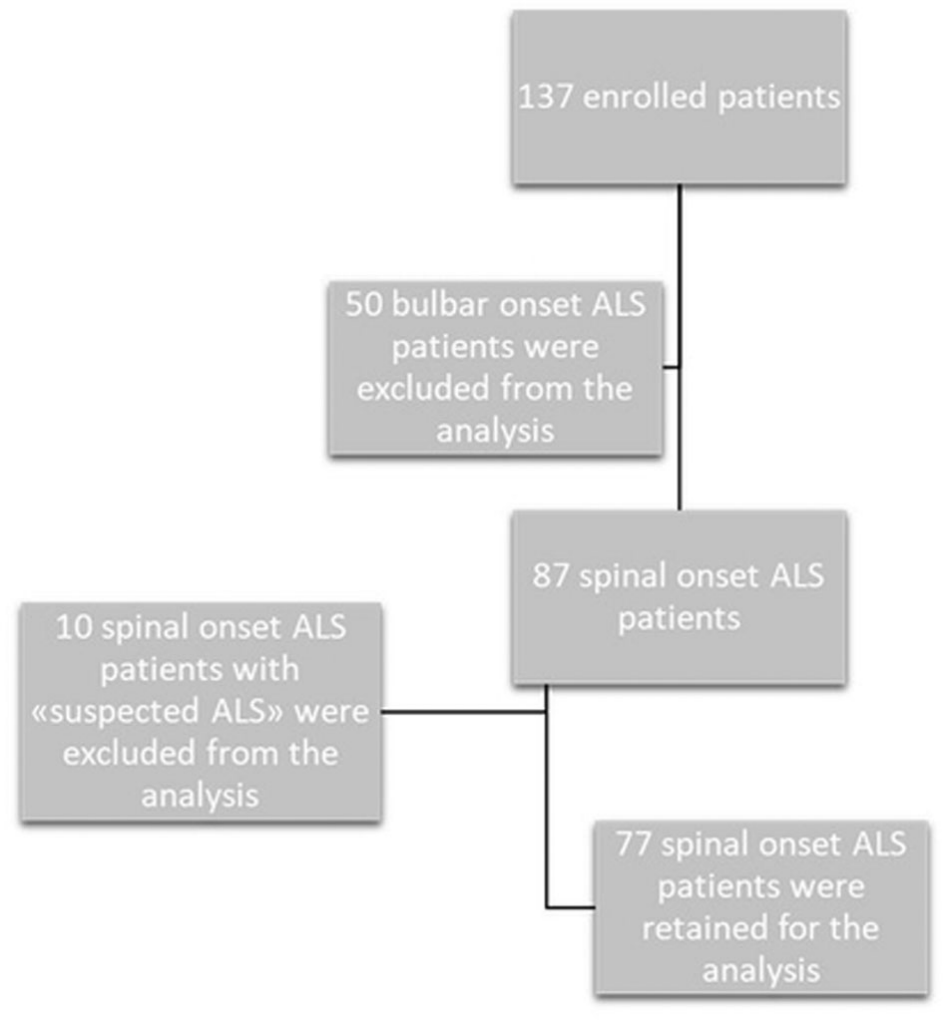
Study population.

Incident ALS patients who were referred to an ALS tertiary center from Apulia Southern Italy, between 2015 and 2021, were consecutively recruited for the study. Demographic characteristics and clinical data have been registered and collected by experienced neurologists of the ALS team. The following demographic and clinical variables were recorded: age at symptom onset, gender, site of onset, disease duration, and clinical phenotypes (Chio et al., 2011). Patients were defined as definite, probable, possible, and suspected ALS according to the El Escorial criteria (Brooks, 1994) and their revised version of Airlie House (Brooks et al., 2000). Considering the intrinsic divergent clinical profile of patients with suspected ALS (Schito et al., 2020) and primary lateral sclerosis (Gordon et al., 2006), patients with pure LMN and pure UMN were excluded from the analysis. Subsequent analyses focused only on patients with clearly identified, lateralized onset of weakness or wasting in an upper or lower limb (Figure 1).

### 2.2 Spreading patterns

Progression of disease was defined by the extension of weakness or muscle wasting beyond the site of onset, established through a combination of anamnestic data and clinical evaluation. Patients were then divided into groups according to the different initial directions of spread, namely horizontal spreading pattern (HSP), vertical spreading pattern (VSP) or crossed spreading pattern. The direction of spread was considered horizontal when the first region of spreading consisted of cervical to cervical contralateral or lumbar to lumbar contralateral. The vertical direction was defined as spread either from the cervical or lumbar region to the ipsilateral leg or arm. The crossed pattern consisted of those patients in whom symptoms spread in a diagonal pattern either from the cervical or lumbar region to the contralateral lumbar or cervical region.

Therefore, we evaluated the different clinical courses of the disease in patients with HSP and VSP. We used time‐to‐generalization as a mid‐intermediate time point along the disease course (Tortelli et al., 2016).

### 2.3 Clinical evaluation

UMN burden was evaluated using the Penn Upper Motor Neuron scale (PUMNS) (Quinn et al., 2020). LMN burden was calculated using the score previously proposed by Devine et al. (2016). This latter score assigned a separate LMN score for each limb, ranging from 0 (no involvement) to 3 (significant, severe involvement), based on the Medical Research Council and muscle wasting.

The sum of LMN and UMN scores, calculated separately for upper and lower limbs, and the total LMN and UMN scores were retained for further analyses.

All patients were functionally evaluated using the ALS Functional Rating Scale‐Revised (ALSFRS‐r), based on 12 items including bulbar (first to third items), upper limbs (fourth to sixth items), lower limbs (seventh to nineth items), and respiratory symptoms (10th to 12th items). For each patient, selective bulbar (ALSFRS‐r‐B), upper (ALSFRS‐r‐UL), lower limbs (ALSFRS‐r‐LL), and respiratory (ALSFRS‐r‐R) subscores were also calculated.

Normally preserved motor function at each site was defined as a full score of 12 points at the site, according to the ALSFRS‐r score. Percentages of normal preservation of the four motor functions (bulbar, upper limb, lower limb, and respiratory) were calculated at the diagnosis. This latter approach to assess the disease severity separately for each district was already performed elsewhere (Fujimura‐Kiyono et al., 2011). The progression rate was calculated using the following formula: (48‐ALSFRSr)/disease durations (months) (Kimura et al., 2006).

### 2.4 Statistical analysis

Demographic and clinical variables were reported as median (along with interquartile range) or frequencies (percentages) for continuous and categorical variables, respectively. Demographic and clinical characteristics were compared using Mann–Whitney *U* test and the Pearson chi‐squared test for continuous and categorical variables, respectively. Wherever there was a statistically significant difference in demographic variables between patients with HSP and VSP, logistic regression was used to calculate the odds ratio and the 95% confidence interval (CI).

Analysis of covariance was performed to evaluate intergroup differences in UMN and LMN burden between HSP and VSP patients. Assumptions of normality, linearity, and homogeneity of variances were verified. Clinical measures were included as dependent variables and study group allocation as the categorical independent variable. In order to avoid the confounding role of the disease severity at the clinical evaluation, ALSFRS‐r and disease duration were included as covariates. Estimated marginal means of clinical measures were plotted with error bars to illustrate the differences between groups using a boxplot graph. Afterward, the above‐mentioned analyses were performed separately in upper and lower limb‐onset ALS patients to evaluate the differences between patients with HSP and VSP in the sum of the LMN and UMN scores of the arms and legs.

The association between number of preserved body districts and direction of spread, namely VSP and HSP, was tested using Glass rank biserial correlation (rg) (Glass, 1966).

A time‐to‐event analysis was performed based on anamnestic history reported by patients during the first visit: the development of bulbar symptoms (if occurred) was used as endpoints. Time to generalization was used for patients presenting with bulbar involvement at the first visit. Otherwise, the time elapsed between disease onset and clinical evaluation (namely disease duration) was used as a period of observation for the patients who did not experience the events. Log‐rank test was used to test for differences between horizontal and vertical spreading pattern groups.

Subjects were enrolled within the “SLAP‐Dem Study,” a population‐based study on rare neurodegenerative disease that was approved by the Ethic Committee for Medical Research at ASL Lecce on May 25, 2017 (number 6). Both patients and their caregivers provided their informed consent. The present study was conducted according to the World Medical Association's 2008 Declaration of Helsinki, the guidelines for Good Clinical Practice, and the Strengthening the Reporting of Observational Studies in Epidemiology (STROBE) statement.

## 3 RESULTS

During the study period, 137 newly diagnosed ALS patients were identified, of which 87 (64%) presented a limb‐onset. At the first visit, all spinal onset ALS patients had at least two limbs involved, and all of them reported a clearly defined spread direction. Ten patients (7% of the entire cohort and 11.5 % of spinal onset ALS) were pure LMN syndromes and were not included in the analysis, as stated above. No cases of pure UMN syndrome were identified. Among the remaining patients with ALS, 60 were identified as classic ALS (78%), five (6%) as pyramidal phenotype, six (8%) as flail arms, and six (8%) as flail legs.

There were 47 (61%) patients with HSP and 30 (39%) with VSP. No other spread directions were reported in our cohort. The clinical and demographic characteristics of the two groups are reported in Table 1. Patients with HSP and VSP did not show any significant differences regarding gender, age at symptom onset, and diagnostic certainty, expressed by El‐Escorial categories. Classic phenotypes exhibited almost the same probability of vertical or horizontal spreading pattern (25 and 35 out of 60 patients with ALS, respectively) and represented the most common phenotype both in patients with VSP and HSP (83% and 74%, respectively). All patients with pyramidal phenotype exhibited a VSP, while flail arm and flail leg phenotypes showed only an HSP (overall *χ* ^2^ = 15.68, *p* = .001). Patients with VSP showed a shorter disease duration compared to patients with HSP (17 months [range: 10−27] vs. 25 months [range: 12−47; *p* = .04]), with no significant differences in disease severity expressed by ALSFRS‐r scores.

**TABLE 1:** Demographic and clinical characteristics of patients with amyotrophic lateral sclerosis (ALS), dichotomized according to the spreading directions

	Total (77 patients)	Vertical spreading pattern (30 patients)	Horizontal spreading pattern (47 patients)	Vertical spreading pattern vs Horizontal spreading pattern
Age (years)	61 (50–71)	61 (55–70)	61 (48–72)	*p* = n.s.
(median, IQR)				
Sex (male/female)	52/25	20/10	32/15	*p* = n.s.
Male (%)	67%	67%	62%	
Disease duration (months)	22 (12–34)	17 (10–27)	25 (12–47)	*p* = .04
(median, IQR)				
El Escorial categories				
(definite/probable/possible)	21/32/24	10/11/9	12/21/15	* p = n.s*.
Definite (%)	27%	33%	23%	
ALS phenotypes:				
classic/bulbar/flail arm/flail leg/pyramidal/respiratory/PLMN/PUMN	60/0/6/6/5/0/0/0	25/0/0/0/5/0/0/0	35/0/6/6/0/0/0/0	*p* = .001
	78%	83%	74%	
(No. of patients)				
Classic (%)				
ALSFRS‐r at clinical evaluation	35 (28–40)	35 (30–40)	34 (27–41)	*p* = n.s
(median, IQR)				
Progression rate				
(median, IQR)	0.59 (0.35–0.87)	0.76 (0.48–1.80)	0.57 (0.35–0.82)	*p* = .03
Estimated Penn Upper Motor Neuron Score*		11.53 (1.14; 9.26–13.81)	6.63 (0.87; 4.88–8.38)	*p* = .001
(mean, standard error, CI)				
Estimated LMN score mean*		4.78 (0.47; 3.83–	6.79 (0.37; 6.07–	*p* = .001
(mean, standard error, CI)		5.73)	7.53)	
Patients with bulbar symptoms	60/17	25/5	35/12	*p* = n.s.
(Yes/no)	78%	83%	75%	
Yes (%)				
Estimated time to generalization (months)				
(median, standard error, CI)	18 (2.86; 12.40–23.59)	9 (4.1; 0.97−17.03)	20 (5.17; 9.85–30.15)	*p* = .01
Preserved districts				
Three districts: No. of patients (%)		2 (6.6%)	12 (25.5%)	*p* = .005
Two districts: No. of patients (%)		6 (20%)	15 (31.9%)	
One district: No. of patients (%)		9 (30%)	10 (21.3%)	
Zero districts: No. of patients (%)		13 (43.4%)	10 (21.3%)	

Abbreviation: CI, confidence interval; n.s. nonsignificant.

* Estimated marginal means and standard error are adjusted for ALSFRS‐r at clinical evaluation and disease duration (ALSFRS‐r at clinical evaluation = 32.79, disease duration = 28.95 months).

**FIGURE 2:**
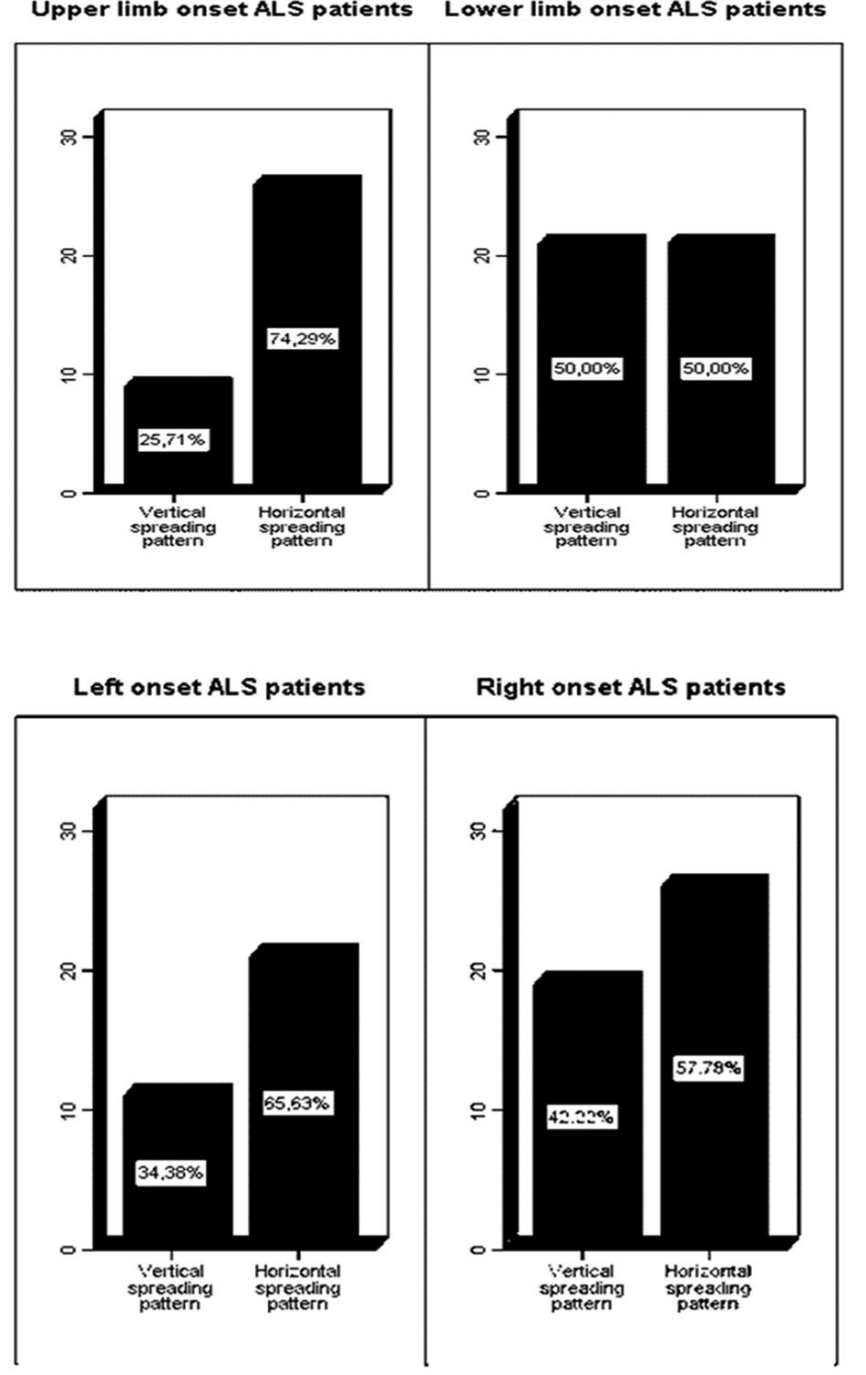
Frequency of the site of the second segment spreading according to the site of onset.

Patients with upper limb‐onset ALS (UL‐ALS) more likely presented an HSP spreading direction (74% vs. 26% VSP; *χ* ^2^ = 4.7; *p* = .03), while the spreading was similar among patients with lower limb‐onset (LL‐ALS) (Figure 2). Therefore, the risk of VSP was 2.9‐fold higher in patients with LL‐ALS compared to patients with UL‐ALS (95% CI 1.01–7.61; *p* = .03). Conversely, no differences in the frequency of vertical/horizontal spreading patterns were found according to the side of symptoms onset (Figure 2).

### 3.1 Clinical differences among HSP and VSP patients

### 3.2 Total population

The clinical profiles of the two subgroups are described in Table 1. Patients with VSP showed a wider overall UMN impairment (estimated marginal mean of PUMNS score 11.53 [95% CI: 9.26–13.81] vs. 6.63 [95% CI: 4.88–8.38; *p* = .001]), compared to patients with HSP. Conversely, patients with HSP exhibited a greater LMN involvement (estimated marginal mean of Devine's score 4.78 [95% CI: 3.83–5.73] vs. 6.79 [95% CI: 6.07–7.53] in VSP and HSP, respectively, *p* = .001).

### 3.3 Upper limb‐onset ALS patients (UL‐ALS)

**FIGURE 3:**
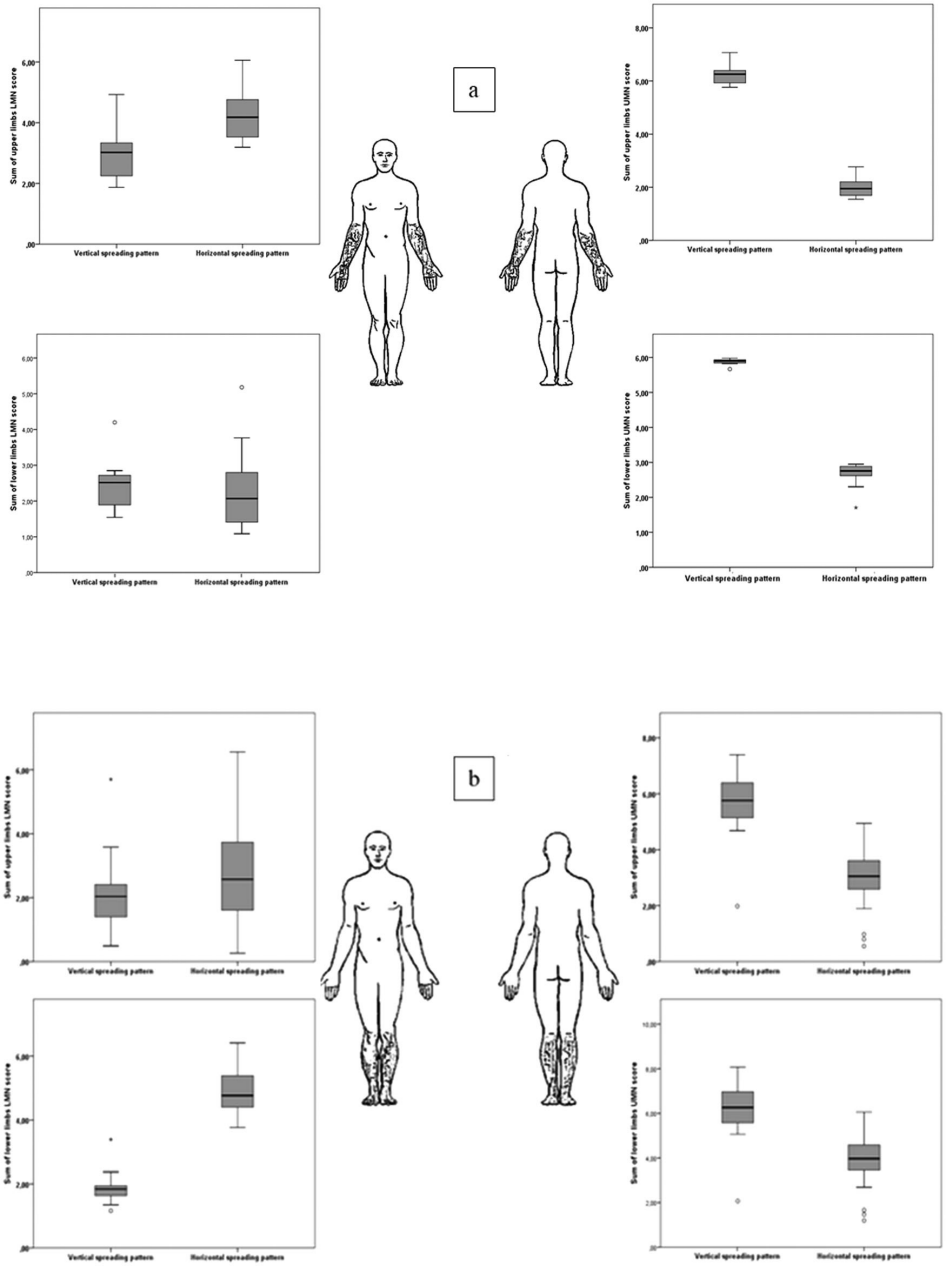
Differences in clinical profiles of patients with amyotrophic lateral sclerosis (ALS) between vertical crossed spreading (VSP) and horizontal spreading pattern (HSP). (a) Upper limb‐onset ALS patients and (b) lower limb‐onset ALS patients.

Among patients with UL‐ALS, those with HSP presented a higher LMN involvement in upper limbs, compared to patients with VSP spread (estimated marginal mean of Devine's score 4.28 [95% CI: 3.61–4.95] vs. 2.71 [95% CI: 1.42–3.99], *p* = .042], while the LMN involvement in lower limbs was similar in the two subgroups. On the other hand, VSP UL‐ALS patients exhibited higher UMN involvement either in cervical (estimated marginal mean of PUMN scores 6.11 [95% CI: 4.09–8.13] vs. 2.04 [95% CI: 0.96–3.05; *p* = .001]) or in lumbar regions (5.78 [95% CI: 3.74–7.83] vs. 2.72 [95% CI: 1.66–3.78; *p* = .01]), when compared to patients with HSP (Figure 3).

### 3.4 Lower limb‐onset ALS patients

LL‐ALS with HSP spread showed higher LMN involvement in the lower limbs compared to patients with VSP (estimated marginal mean of Devine's score 4.71 [95% CI: 4.05–5.35] vs. 2.05 [95% CI: 1.39–2.7; *p* < .0001]), while the involvement of LMNs in upper limbs was similar. LL‐ALS VSP patients exhibited higher involvement of UMN either in cervical (estimated marginal mean of PUMNs score 5.81 [95% CI: 4.47–7.14] vs. 2.84 [95% CI: 1.51–4.17; *p* = .004]) or in lumbar region (6.31 CI [5.04–7.59] vs. 3.73 CI [2.45–5.01; *p* = .008]), compared to patients with HSP (Figure 3).

### 3.5 Assessment of disease severity expressed as ALSFRS‐r score

No significant differences in total ALSFRS‐r score were found between patients with VSP and HSP. However, subgroup analyses revealed that UL‐ALS patients with HSP exhibited a statistically significant lower median value of ALSFRS‐r‐UL subscore, compared to VSP (5 [IQR range: 2–8] vs. 9 [IQR range: 5–10], *p* = .02). Conversely, no significant differences were found in ALSFRS‐r‐B, ALSFRS‐r‐LL, or ALSFRS‐r‐R subscore between HSP and VSP in patients with ALS with the onset in the upper limbs.

LL‐ALS patients with HSP spread showed a statistically significant lower median value of the ALSFR‐r‐LL sub‐score (3 [IQR range: 2–6] vs. 6 [IQR range: 5–8], *p* = .032). Conversely, no significant differences were found in ALSFRS‐r‐B, ALSFRS‐r‐LL, or ALSFRS‐r‐R sub‐score between HSP and VSP in patients with ALS with the onset in the lower limbs.

The number of preserved motor functions, namely bulbar, upper and lower limbs, and respiratory, was significantly higher in ALS patients with HSP compared to VSP (*rg* = 0.321 *p* = .005). Specifically, three or two motor functions were preserved in 25.5% and 31% of ALS patients with HSP, respectively, while only 6.6% and 20% of ALS patients with VSP showed preservation of three or two motor functions, respectively. Conversely, 30% and 41% of ALS patients with VSP showed a wider impairment of three or four motor functions, respectively, compared to 21.3% of ALS patients with HSP.

### 3.6 Progression of disease among with patients HSP and VSP

**FIGURE 4:**
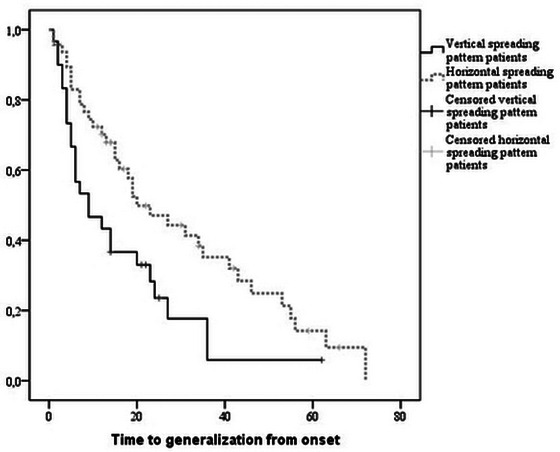
Kaplan Meier survival curves in patients with amyotrophic lateral sclerosis (ALS) stratified according to the spreading directions.

Finally, we investigated the progression of disease in patients with HSP and VSP. Patients with VSP were characterized by a higher median progression rate compared to HSP (0.76 [IQR range: 0.48 −1.80] vs. 0.57 [IQR range: 0.35–0.82]; *p* = .03). At the first visit, only 17 patients among 87 did not report bulbar symptoms, with no statistically significant difference between patients with ALS with the horizontal or vertical spreading pattern. Among all patients with VSP without bulbar symptoms, only five out of 34 exhibited a pyramidal phenotype, while among the 12 patients with HSP without bulbar symptoms, six exhibited a flail arm phenotype, while the remaining six were flail legs. The time‐to‐event analysis revealed that spinal onset ALS patients with VSP exhibited an earlier median bulbar involvement (9 months [range 0.97–17.03] vs. 20 months [range: 9.88–30.12 *p* = .01, Figure 4]), compared to HSP.

## 4 DISCUSSION

In the present study, we found different patterns of the disease spreading among spinal onset ALS patients. The most intriguing findings of our study were the significantly higher prevalence of HSP in UL‐ALS cases and the similar risk of developing VSP and HSP among LL‐ALS patients. Patients with VSP showed a wider impairment of UMN, while patients with HSP exhibited a higher LMN burden. Subgroup analyses performed separately in upper and lower limbs‐onset ALS patients revealed a wider UMN damage in patients with VSP beyond the body district of onset, while LMN impairment was higher in patients with HSP and prominently limited contralaterally to the site of onset. Although the overall disease severity expressed as ALSFRS‐r score did not differ between patients with HSP and VSP, the first ones exhibited more severe impairment in the region of onset (lower score of ALSFRS‐r‐UL and ALSFRS‐r‐LL, respectively, in patients with the onset in upper and lower limbs), while the second ones showed a slighter and wider impairment in several body districts, beyond the site of onset. Consistent with this observation, ALS patients with VSP were characterized by a faster rate of disease progression and the earlier involvement of bulbar muscles.

The theoretical foundations of the present study laid on the most accredited theory of ALS propagation proposed by Ravits et al. in 2014, according to which in patients with ALS, both UMN and LMN impairment were maximal in the same body region. After the disease was triggered and began spreading contiguously, the degeneration proceeded independently at the UMN and LMN levels, delineating the extreme heterogeneity of ALS disease (Ravits, 2014).

Previous studies have retrospectively and prospectively evaluated the spreading direction according to clinical variables (e.g., the site of onset [Fujimura‐Kiyono et al., 2011; Gargiulo‐Monachelli et al., 2012] and the relative impact of UMN and LMN [Gromicho et al., 2020; Körner et al., 2011], this latter expressed also in terms of preserved motor function [Fujimura‐Kiyono et al., 2011] and electromyographic findings [Sekiguchi et al., 2014; Zhenfei et al., 2019]). Conversely, our study aimed to investigate the clinical characteristics of ALS patients according to two different spreading patterns, namely VSP and HSP. Despite this latter methodological difference, our findings confirm the focal onset and clearly lateralized spreading direction of the disease in all patients of our cohort, as reported elsewhere (Brooks, 1991; Körner et al., 2011; Ravits et al., 2007; Ravits et al., 2007). Similar to previous studies (Brooks, 1996; Fujimura‐Kiyono et al., 2011; Körner et al., 2011), motor symptoms spread between adjacent regions either from rostral to caudal and vice versa, following the somatotopically arranged neuroanatomy of the cortex, or horizontally to the adjacent region, following the spinal cord distribution of the LMNs.

None of our patients exhibited “crossed” spreading patterns, although the non‐contiguous pattern of disease spreading has been reported in previous population‐based studies (Gargiulo‐Monachelli et al., 2012; Zhenfei et al., 2019), in a limited number of spinal onset ALS patients (ranging from 8% [Gargiulo‐Monachelli et al., 2012] to 14% [Kimura et al., 2006]). Previous studies have ascribed the “crossed pattern” to the lack of sensitive clinical and functional signs of the thoracic region, whose involvement may have gone unnoticed in patients progressing from lumbosacral to upper limb region and vice versa (Gromicho et al., 2020). Furthermore, Sekiguchi et al. (2014) used electromyography to test “contiguous” versus “multifocal hits” progression in 39 patients, and the authors proposed a “multifocal hits and local propagation” hypothesis. The absence of ALS patients with “crossed pattern” in our cohort may be explained by the relatively small size of our population, which did not allow us to incept this rare non‐contiguous pattern of disease progression.

Another important observation of our study is the higher prevalence of HSP among upper limb‐onset ALS patients. Since VSP seemed to reflect a preferential involvement of the somatotopically arranged neuroanatomy of the cortex, the lower VSP prevalence among patients with UL‐ALS is probably due to the more susceptibility of cortical motor neurons innervating the lower limbs to neurodegeneration. Specifically, the anterograde (or Wallerian) axonal degeneration of cortical motor neurons in ALS disease was previously demonstrated by numerous radiological studies (Agosta et al., 2010; Blain et al., 2011; Filippini et al., 2010), and it followed a neuronal insult, which could occur anywhere along corticospinal tract. Therefore, it could be speculated that longer motor neurons (e.g., those innervating lower limbs) are more susceptible to neuronal damage compared to shorter neurons (e.g., those innervating upper limbs).

Our study also showed that while the classic ALS phenotype could equally exhibit a VSP or HSP, different ALS phenotypes exhibited a preferential spreading pattern. Specifically, the pyramidal phenotype showed exclusively a VSP, while the flail arm and the flail leg exhibited only an HSP. These latter findings agree with previous studies which reported that higher UMN impairment (as found in pyramidal symptoms) was reflected by wider motor and extra motor cortical thinning and lower diffusion tensor imaging metrics of the corticospinal tract (Mezzapesa et al., 2013; Woo et al., 2014). Conversely, flail arm and flail leg phenotypes differ from healthy controls neither in cortical thickness (Schuster et al., 2013) nor in ALS peculiar neurophysiological index (e.g. split hand sign; Yang et al., 2015). Therefore, we agree with the previous authors who speculated that classical ALS and its variant could or could not originate from the motor cortex (Yang et al., 2015), and we suggested to delineate the spreading pattern in order to timely incept rare variants (e.g., pyramidal, flail arm, flail leg phenotypes) with undoubtedly different course of the disease (Schito et al., 2020).

To the best of our knowledge, our study for the first time evaluated the impairment of UMN and LMN according to different spreading directions, using well‐known quantitative scores. A recent previous study adopted an exactly opposite methodological approach, evaluating the spreading directions after dichotomizing patients according to predominant UMN or LMN impairment (Gromicho et al., 2020). Differently from this latter study which reported that the next involved body part was the contralateral limb in both patients with predominant UMN or LMN signs, we observed that VSP has a wider impairment of UMN, while HSP exhibited a higher LMN burden. Our findings confirmed the abovementioned theory according to which VSP and HSP seemed to reflect a preferential somatotopically distribution of UMN and LMNs. To date, only one study evaluated UMN involvement through quantitative scales in patients with different spreading directions (Hu et al., 2016), and the study reported similar results.

Furthermore, we demonstrated that the wider UMN impairment observed in VSP usually oversteps the site of onset even in the early phases of the disease, while the prominent LMN involvement in HSP is largely confined to the region of onset (e.g., cervical or lumbar), at least at the time of diagnosis.

The absence of statistically significant differences among ALSFRS‐r total score between ALS patients with VSP and HSP has been previously reported (Hu et al., 2016). Nonetheless, we demonstrated that patients with HSP exhibited a greater drop of ALSFRS‐r sub‐score (e.g., 3–4 points) in the region of onset, while VSP showed a slighter (e.g. 1–2 points) but more diffuse reduction of ASLFRS‐r subscore in more body districts beyond the site of onset. Since patients with VSP and HSP were characterized by prevalent UMN and LMN, respectively, it could be speculated that the UMN burden is more associated with the onset and spreading of the ALS disease rather than the LMN burden, as confirmed by epidemiological, clinical (Devine et al., 2014), and neurophysiological studies (Zoccolella et al., 2021).

These latter results were corroborated by the evidence that among spinal onset ALS patients, those with VSP (that more likely showed a UMN pattern) exhibited a shorter disease duration and a higher progression rate, and an earlier bulbar involvement, compared to HSP spread. Therefore, patients with VSP reached bulbar involvement and a higher degree of disability early compared to patients with HSP, suggesting a possible broader or even faster clinical course.

A possible explanation of these latter results laid in the neuroanatomical distribution of UMN along about 3−6 cm of cortical motor neurons. Conversely, at the LMN level, the spread first occurs to the contiguous contralateral hand/arm areas, while spread to the ipsilateral foot/leg is far remote through the long thoracic span (about 26 cm) (Ravits & La Spada, 2009).

However, our study has potential limitations as it was a retrospective study conducted on a relatively small sample of consequent patients enrolled in a tertiary ALS center, which could have led to potential selection bias. Another potential limitation, consistent with the large part of available studies, is the chance of error in evaluating the progression pattern on a single point amnestic data in patients with a long progression time. However, we believe however that this chance of error in our study cohort is limited given the overall median disease duration of 22 months.

In conclusion, although preliminary, our findings suggest investigating and underlining the spreading direction of ALS among spinal onset cases, in order to predict an earlier involvement of bulbar symptoms and eventually a more rapid course of the disease.

## AUTHOR CONTRIBUTIONS

**Giammarco Milella**: Conception of the study, statistical analysis, interpretation of data, drafting the initial manuscript, and revising it for important intellectual content. **Stefano Zoccolella**: Conception of the study, statistical analysis, interpretation of data, and drafting the initial manuscript and revising it for important intellectual content. **Daniele Urso**: Acquisition of data, data curation, and reviewed the manuscript for intellectual content. **Salvatore Nigro**: Acquisition of data, data curation, and reviewed the manuscript for intellectual content. **Ludovica Tamburrino**: Acquisition of data, data curation, and reviewed the manuscript for intellectual content. **Valentina Gnoni**: Acquisition of data, data curation, and reviewed the manuscript for intellectual content. **Marco Filardi**: Data curation, interpretation of data, and reviewed the manuscript for intellectual content. **Giancarlo Logroscino**: Conception of the study, study supervision, drafting the initial manuscript, and revising it for important intellectual content.

## CONFLICT OF INTEREST STATEMENT

The authors report no conflict of interest.

## FUNDING INFORMATION

This work has been supported with the funding from Regione Puglia and CNR for Tecnopolo per la Medicina di Precisione: D.G.R. No. 2117 of 21.11.2018 (CUPB84I18000540002)—C.I.R.E.M.I.C. (Research Center of Excellence for Neurodegenerative Diseases and Brain Aging)—University of Bari “Aldo Moro.”

### PEER REVIEW

The peer review history for this article is available at https://publons.com/publon/10.1002/brb3.2899.

## Data Availability

The data that support the findings of this study are available from the corresponding author, upon reasonable request.
